# Judicial bypass for minors post-*Dobbs*

**DOI:** 10.1177/17455057231219601

**Published:** 2023-12-25

**Authors:** Sarah Horvath, Susan Frietsche

**Affiliations:** 1 Department of Obstetrics and Gynecology, Penn State University College of Medicine, Hershey, PA, USA; 2 Women’s Law Project, Pittsburgh, PA, USA

**Keywords:** abortion, adolescent health, judicial bypass, minor, parental involvement laws, reproductive health

## Abstract

State laws that require minors seeking abortion care to notify or obtain consent from a parent or other legal guardian are broadly referred to as parental involvement laws. Judicial bypass allows a minor to petition the court to bypass parental involvement. Even before the *Dobbs v Jackson Women’s Health Organization* decision overturned *Roe v Wade* on 24 June 2022, 36 states had at least one parental involvement law, making minor access to abortion care even more complex than adult access. Since the *Dobbs* decision, at least 15 states have completely banned abortion, adding further complexity, geographic barriers, and inequities to the pursuit of reproductive healthcare. In this narrative review and commentary, we explain parental involvement laws and judicial bypass from both a legal and medical perspective, exploring the evolving challenges created by this system in the year post-*Dobbs.*

## Introduction

Even before the Supreme Court’s *Dobbs v Jackson Women’s Health Organization* decision overturned *Roe v Wade*, 410 U.S. 113 (1973) and *Planned Parenthood v. Casey*, 505 U.S. 833 (1992), on 24 June 2022, accessing abortion care in the United States was complicated.^1–3^ Being under the age of 18 years adds an additional layer of complexity that continues to evolve in the post-*Dobbs* policy environment. Minors’ ability to consent to their own sexual and reproductive healthcare, including contraception, pregnancy, and abortion care, is governed by a patchwork of federal and state caselaw, statutes, and funding regulations, such as the regulations implementing the Title X family planning funding program.^4^ As federal protection of reproductive rights has receded, states have acquired increased authority to control policy in this area. Thus, minors’ access to care is highly dependent upon geography. As of this writing (17 October 2023), 16 states ban all or most abortion for all people, regardless of age.^5^ An additional 21 states require minors to involve parents or legal guardians in their decision to access abortion care.^6^

**Figure 1.**
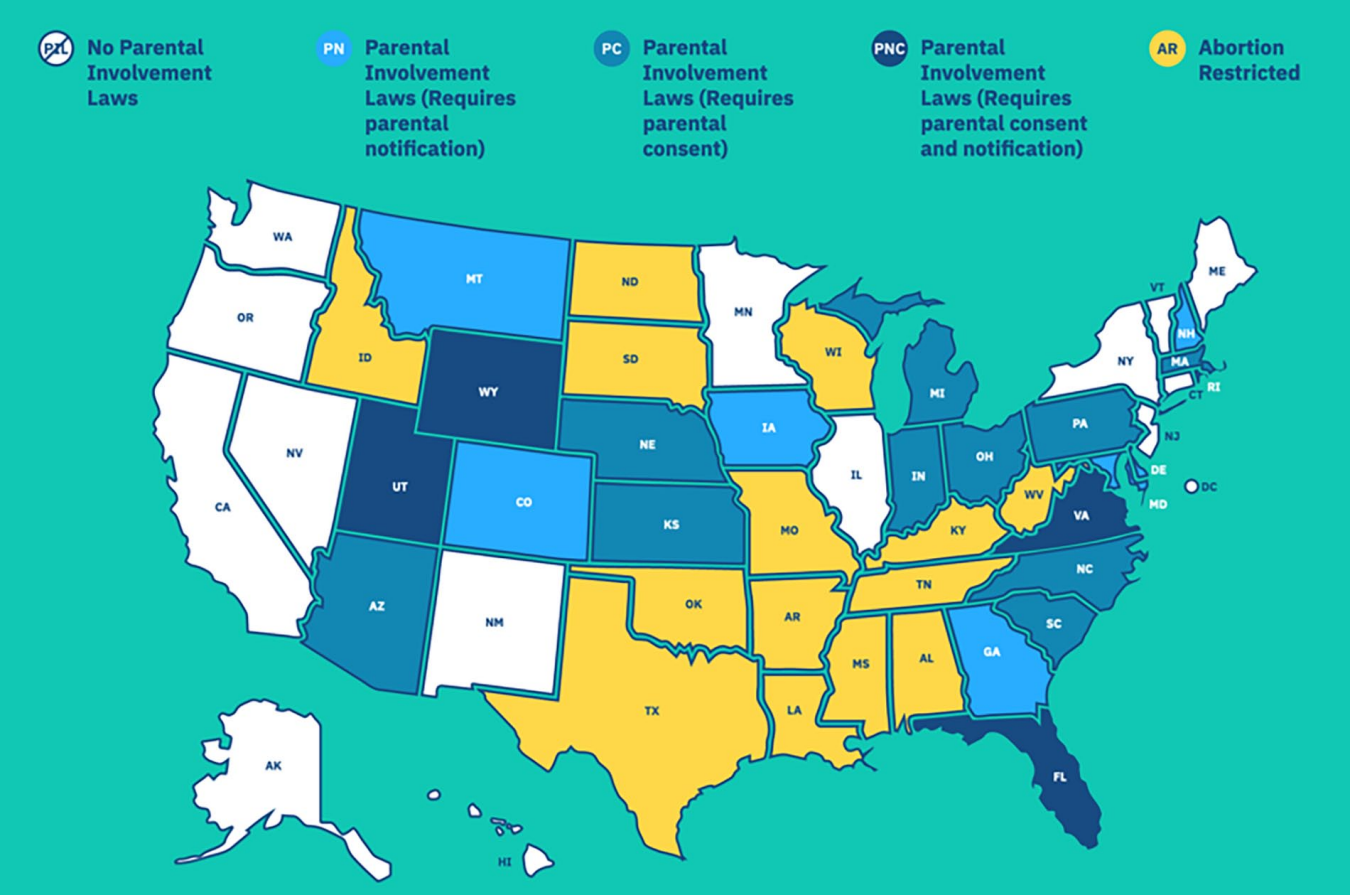
Judicial bypass United States map reprinted with permission from the Judicial Bypass Wiki from if/when/how. Map is current as of October 2023. For an updated, interactive version of this map, please see https://judicialbypasswiki.ifwhenhow.org/.

Parental involvement laws vary by state and apply to the location of the clinic where care will be obtained. (See Table 1 and Figure 1) Legislation may require that a doctor notify a parent or legal guardian in advance of the procedure (notification), or that the patient’s parent or legal guardian provide written and signed consent (consent). Three states require both. The timing and details of these requirements vary and may be difficult to navigate. The requirements most often apply to minors under 18, but some statutes apply only to those under 17 or 16. In some parental involvement states, consent of a doctor, grandparent, or mental health professional can substitute for parental consent. Minors without access to their parents or legal guardians, those with fewer monetary resources, unstable housing or family situations, and those who are victims of incest may have increased difficulty navigating these laws.

**Table 1.** State variation in parental involvement laws (as of October 2023).^6^

State	Notify or consent	Details^a^	Judicial bypass assistance
Arizona	C	Written permission; notarized	Dedicated advocates
Colorado	N	Doctor notifies parent or guardian 48 h in advance	Dedicated advocates
Delaware	N	Doctor notifies parent or guardian 24 h in advance (< 16 years only)	“Judicial waiver” through notarized written request
Florida	Both	Written permission + doctor notifies	Contact clinic
Georgia	N	Doctor notifies parent or guardian 24 h in advance	Contact clinic
Indiana	C	Written permission	Dedicated advocates
Iowa	N	Doctor notifies parent or guardian or grandparent 48 h in advance	Contact clinic
Kansas	C	Counseling session with parent, guardian, or other adult > 21 years; consent of both parents	Dedicated advocates
Maryland	N	Doctor attempts to notify parent or guardian	N/A
Massachusetts	C	Written permission (< 16 years only)	Dedicated advocates
Michigan	C	Written permission	Contact clinic
Montana	N	Doctor notifies parent or guardian 48 h in advance (< 16 years only)	Contact clinic
Nebraska	C	Written permission	ACLU-NE
New Hampshire	N	Doctor notifies parent or guardian	Dedicated advocates
North Carolina	C	Written permission	Dedicated advocates
Ohio	C	Written permission	Contact clinic
Pennsylvania	C	Written permission	Dedicated advocates
Rhode Island	C	Written permission 48 h in advance	Contact clinic
South Carolina	C	Written permission from parent, guardian, grandparent, *in loco parentis* (< 17 years only)	Contact clinic
Utah	Both	Written permission; Doctor notifies parent or guardian 24 h in advance	Dedicated advocates
Virginia	C	Written permission	Contact clinic
Wyoming	Both	Written permission; Doctor notifies parent or guardian 48 h in advance	Contact clinic

a Except where otherwise noted, “written permission” means written permission from one parent or legal guardian.

When a minor is unable to involve their parent or guardian in their decision to have an abortion, they may petition for a legal exemption through judicial bypass. Judicial bypass is the process of asking a judge to grant the pregnant minor the right to obtain an abortion without the state-mandated parental involvement. The specific process, timing, and criteria for obtaining judicial bypass vary by state.^6^ Here, we describe the evolution and current reality of judicial bypass, the legal and medical consequences of navigating parental involvement laws, and the changes occurring to the system following the *Dobbs* decision.

## Legal framework for judicial bypass

A common feature of parental involvement laws is a judicial bypass alternative: a confidential legal proceeding where a judge grants a mature minor full capacity to consent to their own abortion care, or, if she is not mature, where the judge determines if abortion is in the minor’s best interests. In a line of cases beginning with *Planned Parenthood of Central Missouri v. Danforth*, 428 U.S. 52 (1976), and *Bellotti v. Baird*, 443 U.S. 622 (1979), the US Supreme Court determined that minors have constitutional privacy rights protecting decisions around abortion and other reproductive health care.^7,8^ While the Court noted that the rights of young people are not fully co-extensive with the rights of adults, it recognized that mature minors are capable of making their own decisions about whether to continue a pregnancy, and that no third party, not even a parent, should be given veto power over the minor’s decision. *Bellotti* established the framework for judicial bypass rulings: if, after a hearing at which a minor is represented by counsel, a judge determines that the minor is well informed and mature, or alternatively that an abortion would be in the minor’s best interests, the judge must grant the minor’s petition.

Judicial bypass hearings are unlike most court hearings. Bypass hearings are *ex parte*: that is, there is no opposing party to argue against the minor petitioner; nonetheless, they can become contentious if the judge assumes the role of inquisitor. Bypass hearings are typically conducted in closed courtrooms and with anonymous pleadings that may be filed under seal to protect the minor’s privacy. They are required to be expedited: pregnancy is time-limited and accessing abortion care becomes more complex with increasing gestational age.^9^ For a court proceeding to offer any meaningful relief to a bypass petitioner, it must be prompt. Despite the mandate that the judicial bypass process will be confidential, expeditious, and non-adversarial, the reality can look very different.

First, the bypass hearing threatens the minor’s privacy. As a general rule, bypass hearings take place in person at courthouses, with the exception of a few jurisdictions that have continued to offer virtual hearings innovated during the COVID-19 pandemic. The more likely appearance in court inevitably exposes bypass petitioners to the risk of being recognized by court personnel and other litigants. In addition, courts are typically open during standard business hours, when most minors are required to be in school or at other monitored activities. Repeated absences to meet with a lawyer, attend the hearing, and go to medical appointment(s) create a danger of discovery. In addition, unexcused school or work absences may have negative repercussions on grades, participation on sports teams and other activities, or employment, well beyond the threat to privacy.

Second, almost all bypass hearings delay the minor’s abortion care. The schedules of the courts, bypass attorney, healthcare clinics, and minor may not align, adding unintentional delay. Studies of judicial bypass proceedings have found many courts unwilling or unable to assist a minor petitioner gain access to a bypass hearing.^10^ One recent study of judicial bypass in Ohio found that nearly two-fifths of Ohio courts refused to or could not answer any questions about the judicial bypass process.^11^ The same study found that Ohio’s abortion law was so ill-defined that even attorneys were unsure of which provisions were in effect, and abortion providers were unable to help their patients negotiate their way through the process.

No matter how quickly a court is willing to schedule a bypass hearing, the logistics involved with arranging transportation to court and to the medical provider, getting excused from school or work, possibly arranging for childcare, and raising the fee for the abortion are daunting and can add days or weeks of further delay. Therefore, minors may “time out” of being able to access medication abortion, care at their nearest clinic, or any care at all.^12^ Cost and complexity of care may also increase with increased delays, creating a cycle wherein the delays associated with initially obtaining care lead to new barriers that create further delay.^13^

Finally, the bypass process is a test of the young petitioner’s nerve, as their fate depends on whether they successfully convince a judge that they are mature enough to make an autonomous decision or that an abortion would be in their best interests. Even in the best of circumstances, with the kindest lawyer and the friendliest judge, such a power dynamic may feel adversarial. It is not surprising that many bypass petitioners report that the court hearing caused far more anxiety than the abortion itself. This anxiety is often justified: minors in one study by Coleman-Minahan et al.,^14^ experienced the judicial bypass process as a traumatizing form of punishment for their sexuality, pregnancy, and abortion decision. This study concluded that “the bypass process itself causes emotional harm through unpredictability and humiliation. Despite participants’ resilience, the process may have negative consequences for adolescent health.”

While no national statistics exist on the rate at which judicial bypass petitions are denied, a recent study of judicial bypass in Florida and Texas determined that from 2018 to 2021, denials increased in Florida from 6% to 13% and remained stable in Texas at 5%–7%.^15^ In Pennsylvania, bypass petitions have been denied because the judge believed that the petitioner’s pregnancy was too far advanced, despite still being within Pennsylvania’s gestational age limit; because the judge decided that the minor’s decision not to involve her parents was evidence of immaturity; and because the petitioner made two grammatical errors during her hearing.^16,17^ These petitioners ultimately received the abortion care they sought despite the denial of their petitions, either by traveling to a jurisdiction without a parental consent law or by winning an expedited appeal in a higher Pennsylvania court. These minors did not respond to the denial of their petition by involving a parent or continuing the pregnancy. The net result of the parental consent with judicial bypass law, even for those minors deemed too immature to make their own decisions, was simply to delay their care by forcing them to travel farther from their home or await an appellate court reversal of the trial court’s error.

## Medical consequences of the judicial bypass process

Judicial bypass increases the time to access abortion care. In one study by Janiak et al.,^12^ including 1559 (77%) abortions obtained with parental consent and 467 (23%) using judicial bypass, time from initial contact to abortion care was 8.6 days for those obtaining parental consent and 14.2 days for those using judicial bypass. After adjusting for confounders, minors who used judicial bypass had a 5.2 day longer time between initial contact with the clinic and obtaining care. Another study found that judicial bypass added an average of 6.4 days prior to clinic appointment.^18^ These studies were conducted in Massachusetts and Illinois, both states with robust support networks for minors navigating the judicial bypass process and, thus, likely underestimate the burden in other states. While abortion care is safe at any gestational age, and always safer than term delivery, there is a minimal increase in risk with each week of gestation and laws that cause artificial delay go against best medical evidence.^13,19^

The adjusted odds of becoming ineligible for medication abortion due to increased delay with judicial bypass were 1.57 in the Janiak study.^12^ This is an important finding, as patients may preferentially choose medication abortion over a procedure for myriad personal reasons, including a perception that medication abortion is safer or more natural, enhances privacy, allows them to be in the home setting, have more control over the process, or for avoiding a procedure.^20,21^ Patients with a history of rape, incest, or other trauma may particularly prefer to avoid a procedure and the Trauma Informed Care approach includes maximizing patient safety, choice, and privacy.^22^ Parental involvement laws and the judicial bypass process are antithetical to a trauma informed approach to care.

Mandated parental involvement laws can put the minor at other health risks, such as family violence, abuse, coercion, or rejection.^23^ Minors overwhelmingly involve a trusted adult in the abortion care decision, but that adult may be another family member or trusted mentor, rather than a parent.^24,25^ One-third of those who choose not to involve parents have already experienced violence and fear recurrence.^25^ The American Academy of Pediatrics’ (AAP) 2022 policy statement outlines the importance of supporting adolescent autonomy, noting that most minors involve trusted adults or parents in their abortion decision regardless of a legal mandate to do so. The AAP encourages open communication with parents but opposes mandated parental involvement.

Importantly, abortions after judicial bypass are more common among minors identifying as Hispanic, non-Hispanic black, or other race, those of low socio-economic status (as indicated by having Medicaid insurance) and those with a prior birth or prior abortion (all *p* < .05).^12,26^ Thus, parental involvement laws deepen inequities in access to abortion care and are opposed by the American College of Obstetricians and Gynecologists (ACOG).^19^ There is some evidence that minors will travel out of state to avoid having to comply with parental involvement laws, some because they do not know about the judicial bypass process.^27,28^ Those compelled to travel longer distances, arrange time away from school or family, or navigate complex judicial bypass requirements may be forced to carry their pregnancies to term.^23^ Structural inequities put Black, Indigenous, and People of Color, (BIPOC) and immigrant adolescents at higher risk of experiencing barriers, in direct opposition to the reproductive justice framework, which states that all people should have the right to have a child, the right to not have a child, and the right to parent a child or children in safe and healthy environments.^29^

## Changing legal landscape post-*Dobbs*

The *Dobbs* decision brought the loss of federal abortion rights, thus allowing each state to pass bans or restrictions individually. In the year since *Dobbs*, the legislative map has changed every few weeks, subject to new legislation, elections, ballot measures, and legal decisions.^5^ This, coupled with national-level headlines about mifepristone availability, legislative proposals, and political rhetoric, make abortion care confusing to navigate for people of all ages. Even in states where abortion remains available, appointments are more difficult to obtain given the increase in patients traveling from states that have restricted or banned abortion.^30,31^ Because adolescents have unique barriers to travel, the fallout from *Dobbs* has been especially devastating for them.

The fall of *Roe* and *Casey* raises the question of whether the judicial bypass alternative required by *Bellotti* will remain a feature of parental involvement schemes now that abortion is no longer a protected right under the federal Constitution. In several states, state constitutional and statutory protections are filling the void created by *Dobbs.* Litigators are developing theories to support reproductive autonomy that are not dependent on the constitutional reasoning of *Roe* and *Casey* and that are not foreclosed by *Dobbs*: for example, theories grounded in the Privileges or Immunities Clause of the Fourteenth Amendment, the Ninth Amendment, or equal protection doctrine. Whether these alternative approaches will fully protect minors’ access to abortion, and whether judicial bypass will remain a feature of parental involvement laws, is yet unknown. Quinter and Markowitz^32^ argue that the right to bodily integrity, the “mature minor doctrine,” and numerous other state laws allowing minors to consent for some medical care, support a continuing legal basis for judicial bypass. The need for judicial bypass in states with access to abortion has likely increased, but to a lesser extent than expected. This may be due to minors’ greater reliance on self-managed abortion, undesired or unsafe disclosure to parents, greater numbers of minors carrying to term against their will, or some combination thereof.

The specific legal risks associated with self-managed abortion and interstate provision of medication abortion are developing every day and are beyond the scope of this article; however, minors may access self-managed abortion at higher rates than older patients, so clinicians should have an awareness of the practice.^33,34^ The confusion, lack of uniformity, and fog of potential criminal liability surrounding safe abortion and maternity care threaten providers and harm patients. The stigma surrounding abortion has led to the denial of critical obstetric care as hospitals have turned patients away out of fear of criminal liability if they complete a miscarriage. (*See Zurawski v. State of Texas*, litigation brought by five women whose lives were put at risk after being denied the lifesaving abortion care they needed in their home state of Texas.)^35^ It has also led to medical professionals breaching patients’ Health Insurance Portability and Accountability Act (HIPAA)-protected medical privacy rights by inappropriately—indeed, illegally—reporting suspected abortions to law enforcement authorities.^36^ For minors, HIPAA may also be violated by healthcare practitioners’ illegal disclosure to parents or other family members, and potentially put patients in harm’s way.

Proposals once thought too extreme to be taken seriously are proliferating, including more severe variations of parental consent statutes. In Idaho, for example, a new law which, as this article goes to press, has been temporarily enjoined, creates the crime of “abortion trafficking” and criminalizes “recruiting, harboring, or transporting” minors to help them access abortion without parental consent.^37^ Because they are politically vulnerable, minors are an appealing target for the next wave of restrictions on abortion and contraception.

Despite the *Dobbs* Court’s pronouncement that it was returning abortion policy to the states, ambitious efforts to criminalize abortion nationally are underway. In *Alliance for Hippocratic Medicine v. FDA*, anti-abortion doctors (and a dentist) have challenged the FDA’s approval of mifepristone, the first of two medications commonly used in medication abortion. If they succeed in removing it from the market, they will not only impact the most common method of abortion care, but the evidence-based regimen for miscarriage management, as well. Their lawsuit also seeks to revive the long-dormant Comstock Act, an 1873 federal law criminalizing sending obscene materials by US mail or common carrier and expanding the definition of obscenity to include any materials for preventing conception or producing an abortion.^38^ Until recently, the thought that such a relic could be enforced today was unimaginable. Regardless of the outcome of this particular case, we are likely to continue to see new assaults on the evidence-based practice of reproductive medicine.

These trends threaten several bedrock American freedoms in addition to reproductive autonomy, including First Amendment rights of free speech and association, equal protection guarantees, and the fundamental right to travel. As supporters of reproductive freedom counter restrictions on reproductive rights, they should focus special attention on minor patients, who are increasingly the targets of abortion opponents. Even prior to *Dobbs*, many providers identified heightened legal concerns about providing care to minors. These concerns are amplified by the relative anonymity provided by telehealth.^39^ Telemedicine abortion care became more widely available during the COVID-19 pandemic and should be accessible by minors as well as adult patients, given the geographic inequities of abortion access.^40,41^

## Resources for minor access to care

Minors can access updated, state-specific information on parental involvement laws and the judicial bypass process through non-profit organizations, such as If/When/How by going to their website https://judicialbypasswiki.ifwhenhow.org. This wiki also provides access to a judicial bypass helpline, through the phone or online and serves as a repository for other abortion care resources (see Figure 1).

Online resources such as https://www.ineedana.com/ and https://www.abortionfinder.org/ can direct patients to clinics by geography and provide information on applicable laws for minors. The resource https://www.plancpills.org/ is an aggregator for sourcing abortion pills, by state. It provides information on legality of abortion in general and via telemedicine by location. It also connects to online sources for purchasing mifepristone and misoprostol. The resource https://abortionfunds.org/ is a network of local organizations that can assist with navigating access to abortion care, including costs, by geography.

## Conclusion

Parental consent and notification laws complicate abortion care access and harm minor patients. Judicial bypass can either exacerbate or mitigate (but never eliminate) the harms of parental involvement laws, as it delays care, deepens inequities, and creates unique stigma for adolescent patients. Post-*Dobbs*, these laws have increased in complexity, even as many minors attempt to navigate interstate travel to access care. Some states, such as Idaho, are now attempting to criminalize their citizens’ ability to travel for legal care. Professional organizations, such as the AAP and ACOG, continue to oppose parental involvement laws given the harms that they cause. Clinicians, researchers, and advocates can promote minor patients’ health by protecting their confidentiality, understanding local legislative requirements and resources, supporting judicial bypass attorney networks, and advocating for true policy change that would revoke the burdensome requirement of parental involvement.

## Declarations

**Ethics approval and consent to participate:** Not applicable, as this is a review of current literature and commentary piece.

**Consent for publication:** Not applicable, as no participants were recruited.

**Availability of data and materials:** Not applicable, as the materials are confined to the references, which are published herein.
